# Ecological significance of dark carbon fixation driven by ammonia oxidation in estuarine waters

**DOI:** 10.1016/j.eehl.2026.100258

**Published:** 2026-06-15

**Authors:** Bolin Liu, Xinyu Wang, Can Jiang, Lin Qi, Qianqian Bi, Guoyu Yin, Hongpo Dong, Xiaofei Li, Xia Liang, Ping Han, Min Liu, Yanling Zheng, Lijun Hou

**Affiliations:** a State Key Laboratory of Estuarine and Coastal Research, Yangtze Delta Estuarine Wetland Ecosystem Observation and Research Station, East China Normal University, Shanghai, 200241, China; b Key Laboratory of Geographic Information Science, School of Geographic Sciences, Ministry of Education, East China Normal University, Shanghai, 200241, China; c School of Energy and Environment and State Key Laboratory of Marine Environmental Health, City University of Hong Kong, Hong Kong, China

**Keywords:** Dark carbon fixation, Ammonia oxidation, Microbial communities, Chemoautotrophs, Nitrifiers, Yangtze estuary

## Abstract

Microbial-mediated dark carbon fixation (DCF) is increasingly recognized as an important process for primary production in global oceanic ecosystems, with ammonia oxidation (AO) providing a major energy source for DCF in oxygenated waters. However, a significant research gap remains in integrating the DCF and AO processes mediated by ammonia-oxidizing archaea (AOA) and bacteria (AOB) into a systematic framework within estuarine ecosystems. In this study, we investigated the DCF and AO rates in water samples from the Yangtze Estuary through incubation experiments with ^14^C and ^15^N isotope probing techniques. The measured DCF and AO rates ranged from 17.58 to 168.70 nM C/h and from 4.78 to 499.63 nM N/h, respectively. Our results revealed significant positive correlations among DCF rates, AO rates, and ammonium concentrations. Amplicon sequencing revealed that chemoautotrophs utilizing the Calvin–Benson–Bassham (CBB) cycle were predominantly affiliated with Burkholderiales, while those utilizing the 3-hydroxypropionate/4-hydroxybutyrate (3-HP/4-HB) cycle were primarily associated with Nitrosopumilaceae. Temperature played a crucial role in shaping the composition of chemoautotrophic communities harboring the CBB cycle, whereas salinity was a key factor modulating the composition of chemoautotrophic communities harboring the 3-HP/4-HB cycle. Niche differentiation within nitrifier communities enables their effective adaptation to the frequent environmental fluctuations characteristic of estuarine ecosystems. This study highlights the ecological significance of DCF driven by ammonia oxidation in estuarine waters and emphasizes the need to integrate DCF into blue carbon assessments in coastal ecosystems.

## Highlights

• DCF process yields 2.9%–58.6% of phytoplankton productivity in estuarine waters.
• Nitrifiers represented by AOA and AOB are key contributors to the DCF process.
• Temperature and salinity mainly affect the CBB and 3-HP/4-HB cycles, respectively.
• Niche differentiation enables nitrifiers to adapt to estuarine environments.

## Graphical abstract

**Figure:**
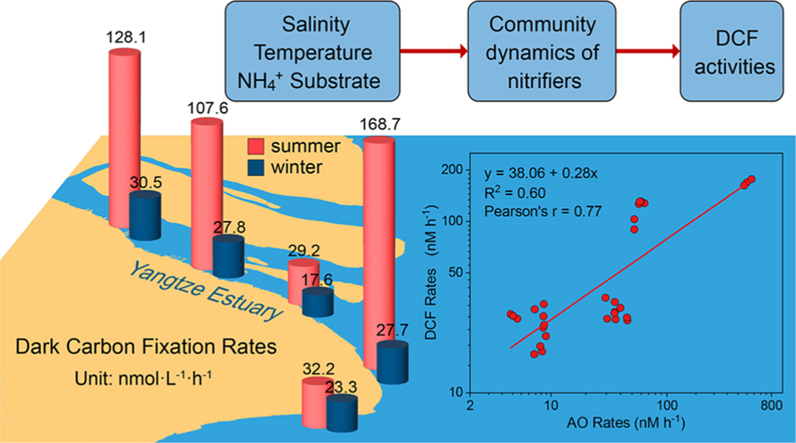

## 1 Introduction

The rising concentration of atmospheric carbon dioxide (CO_2_), a major driver of global warming, represents a pressing challenge that requires more attention from humanity. The need to reduce CO_2_ emissions and enhance atmospheric carbon removal has prompted the development of natural climate solutions including biological processes in the marine environment [1]. Estuarine and coastal ecosystems, which form dynamic interfaces between terrestrial and marine environments, play a disproportionately significant role in global organic carbon burial and sequestration [2]. Accordingly, research and conservation efforts targeting these “blue carbon” ecosystems are increasingly regarded as some of the most critical and cost-effective strategies for mitigating climate change [3]. Dark carbon fixation (DCF), mediated by chemoautotrophic microorganisms, contributes up to 328 Tg C per year in estuarine and coastal ecosystems [4]. In addition to supplying a substantial quantity of organic matter to local food webs, DCF can recapture and recycle respired CO_2_, thereby potentially contributing to carbon retention and sequestration [5,6]. However, DCF has historically been underrepresented in blue carbon assessments of estuarine and coastal ecosystems, largely because of the methodological challenges associated with tracing, quantifying, and evaluating its contribution to CO_2_ sequestration [3,7]. Growing recognition of its ecological importance has led to increasing calls for DCF to be incorporated into estimates of marine primary production and blue carbon cycling [6].

Unlike photosynthetic algae and cyanobacteria that rely on light energy, chemoautotrophic microorganisms obtain energy by oxidizing diverse reduced substrates such as ammonia, nitrite, and sulfide, and subsequently use this energy to fix CO_2_ into organic carbon. Numerous studies have shown that nitrifiers are major contributors to chemoautotrophic carbon fixation in oxygenated aquatic environments [4,8,9]. Nitrification process is generally mediated by either canonical ammonia oxidizers, including ammonia-oxidizing archaea (AOA) and ammonia-oxidizing bacteria (AOB), together with nitrite-oxidizing bacteria (NOB), or by complete ammonia oxidizers (comammox) that can independently oxidize ammonia to nitrate [10,11]. Ammonia oxidation (AO), the initial and rate-limiting step of nitrification, is catalyzed by ammonia monooxygenase, rendering AOA and AOB key contributors to the DCF process within aquatic ecosystems [12,13]. Among these functional groups, AOA utilize the 3-hydroxypropionate/4-hydroxybutyrate (3-HP/4-HB) cycle for carbon fixation, AOB utilize the Calvin-Benson-Bassham (CBB) cycle, and comammox *Nitrospira* utilize the reductive tricarboxylic acid (rTCA) cycle [14,15].

Estuarine waters are characterized by pronounced spatial and temporal environmental variability, driven by the mixing of riverine and marine waters and by complex hydrodynamic processes [16]. These gradients strongly influence microbial diversity and regulate the migration and transformation of both biotic and abiotic components [17]. In estuarine ecosystems, the abundance of canonical AOA and AOB is typically one to two orders of magnitude higher than that of comammox *Nitrospira* [18,19]. Although AO process has been widely investigated in estuarine waters, comparatively few studies have examined DCF in these systems [[20], [21], [22]]. More importantly, the coupling between DCF and AO mediated by canonical AOA and AOB remains poorly integrated within a systematic framework, particularly across strong estuarine gradients. The ecological adaptation and niche differentiation of these ammonia oxidizers also remain insufficiently understood, limiting our ability to evaluate their contribution to coastal blue carbon cycling. The Yangtze Estuary is a typical tide-dominated estuary covering approximately 8500 km^2^. Annually, 2–5 million tons of organic carbon and 2.2 million tons of reactive nitrogen are transported to the estuary [23,24], making it a dynamic transition zone characterized by intense redox activity. In this study, we investigated potential DCF and AO activities and the associated chemoautotrophic microbial communities in the Yangtze Estuary. Specifically, we aimed to: (i) determine the distribution patterns of DCF and AO rates in estuarine waters using ^14^C and ^15^N isotope probing techniques; (ii) characterize the diversity and distribution of functional genes associated with DCF and AO processes; and (iii) evaluate the ecological adaptation of chemoautotrophs involved in the DCF process and their potential significance for coastal blue carbon cycling.

## 2 Materials and methods

### 2.1 Sample collection and environmental parameters

In this study, five sampling sites spanning the freshwater to brackish gradient of the Yangtze Estuary were selected: Liuhekou (LHK), Wusongkou (WSK), Bailonggang (BLG), Chaoyang (CYNC), and Luchaogang (LCG) (Fig. S1). Surface water samples were collected using a pump in winter (February 2021) and summer (July 2021) for DCF and AO rate measurements and microbial analyses. Water temperature, salinity, pH, and dissolved oxygen (DO) were measured *in situ* using a multi-parameter sensor (YSI6920-M, YSI, USA). For nutrient analyses, water samples were filtered through 0.45 μm filters, stored at −20 °C, and analyzed for ammonium (NH_4_^+^), nitrite (NO_2_^−^), nitrate (NO_3_^−^), phosphate (PO_4_^3−^), and silicate (SiO_3_^2−^) using a continuous-flow nutrient analyzer (SAN plus, Skalar Analytical B.V., the Netherlands). Dissolved organic carbon (DOC) and dissolved inorganic carbon (DIC) were measured using a TOC-TN analyzer (TOC-5000, Shimadzu, Japan), with calibration performed using reference samples provided by the Dennis A Hansell lab [25].

### 2.2 ^14^C-DIC incorporation experiments

DCF rates were determined based on the incorporation of radiolabeled bicarbonate (NaH^14^CO_3_; specific activity 57.4 mCi/mmol, PerkinElmer, USA) during dark incubations [26,27]. Water samples were collected directly into 50-mL sterile serum vials after threefold overflow rinsing and the vials were immediately sealed with butyl rubber septa and aluminum caps. A 10-mL helium headspace was then created to minimize atmospheric contamination and facilitate tracer and inhibitor addition. For each sample, triplicate live treatments and one formaldehyde-killed control were incubated in the dark at *in situ* temperature for 24 h. To test the linearity of ^14^C uptake by chemoautotrophs in estuarine waters, time-series incubations were performed for 6, 12, 24, 48, and 72 h (Fig. S2). To differentiate the contribution of nitrifiers to DCF, parallel incubations were amended with NaH^14^CO_3_ and allylthiourea (ATU). ATU is widely used as an inhibitor of ammonia monooxygenase in AOB and has minimal effects on AOA at a concentration of 10 μM [28]. Incubations were terminated by adding formaldehyde to a final concentration of 4%. After incubation, samples were filtered through 0.22 μm sterile filters (Millipore, Bedford, USA). The filters were placed in scintillation vials and exposed to HCl fumes overnight to remove residual inorganic ^14^C. Scintillation cocktail (Ultima Gold, PerkinElmer, USA) was then added to each vial (4 mL), and radioactivity was measured using a liquid scintillation counter (300SL, Hidex, USA). DCF rates were calculated using the following equation:

$$R_{DCF}=\frac{{DPM}_{inc}\times DIC\times 1.05}{{DPM}_{0}\times T}$$

Where, ${DPM}_{inc}$ is the difference in radioactivity (disintegration per minute; DPM) between live samples and killed controls; 1.05 is the isotope coefficient used for correcting the uptake of ^14^C; *DIC* is the ambient DIC concentration (nM); ${DPM}_{0}$ is DPM counts of the added ^14^C-labeled sodium bicarbonate; *T* is the incubation duration (h).

### 2.3 ^15^N-ammonium incubation assay

AO rates were measured using the ^15^N-labeling method with ^15^NH_4_Cl (99 atom% ^15^N, Shanghai Engineering Research Center of Stable Isotope, China) [19]. Water samples were collected into 120-mL sterile serum vials after threefold overflow rinsing and were sealed immediately. A 10-mL helium headspace was created, and triplicate incubations were conducted in the dark at *in situ* temperature for 24 h after adding ^15^NH_4_Cl (≤20% of ambient NH_4_^+^ concentration). To differentiate the contribution of nitrifiers, parallel incubations were amended with ^15^NH_4_Cl and ATU. After incubation, samples were filtered through 0.22 μm sterile filters, and the filtrates were frozen at −20 °C for subsequent analyses. One aliquot of filtrate was used to measure the concentration of NO_x_^−^ (NO_3_^−^ + NO_2_^−^), the oxidation products of AO process. The remaining aliquot was used to determine the δ^15^N of the NO_x_^–^ using the denitrifier method [19]. AO rates were calculated based on the accumulation of ^15^N in NO_x_^–^ pools using the following equation:

$$R_{AO}=\frac{\Delta \left(R_{t}\times {\left[{NO}_{x}^{-}\right]}_{t}\right)}{F_{{\text{NH}}_{4}^{+}}^{15}\times T}$$

Where, $\Delta \left(R_{t}\times {\left[{NO}_{x}^{-}\right]}_{t}\right)$ is the net production rate of ^15^NO_x_^–^ during the incubation; $R_{t}$ is the ^15^N atom ratio in NO_x_^–^ pools; $\left[{NO}_{x}^{-}\right]$ is the concentration of NO_x_^–^ (nM); $F_{{\text{NH}}_{4}^{+}}^{15}$ is the fraction of ^15^NH_4_^+^ at the start of the incubation; *T* is the incubation duration (h).

### 2.4 DNA extraction and quantitative PCR

A 1–2 L aliquot of each sample was filtered through 0.22 μm sterile polycarbonate filters. Genomic DNA was extracted using the DNeasy PowerWater DNA Kit (QIAGEN, Germany) following the manufacturer’s instructions. DNA concentration and quality were determined using a ND-2000 spectrophotometer (Thermo Fisher Scientific, USA) and 1% agarose gel electrophoresis. Functional marker genes encoding RubisCO (Ribulose-1,5-bisphosphate carboxylase/oxygenase; CBB cycle) and ACCase (Acetyl-CoA carboxylase; 3-HP/4-HB cycle) enzymes in chemoautotrophs were quantified using specific primer pairs: cbbL_K2f/V2r (*cbbL*), cbbM-f/r (*cbbM*), and Crena_529F/981R (*accA*). Additionally, primer pairs Arch-amoAF/R and amoA-1F/2R were used to quantify the ammonia monooxygenase genes (*amoA*) of AOA and AOB, respectively. Triplicate DNA samples were quantified using an ABI7500 Fast qPCR System (Applied Biosystems, Canada). Standard curves for qPCR approaches were constructed by tenfold serial dilutions of plasmid standards containing amplicon sequences of the target genes. Only qPCR results with standard curve correlation coefficients > 0.98, amplification efficiencies > 90%, and single melting curve peaks were retained. Details on primers and amplification conditions are provided in Table S1. Sequence reads are available in the National Center for Biotechnology Information (NCBI) Sequence Read Archive (SRA) Database with the project number PRJNA1260199.

### 2.5 Amplicon sequencing and phylogenetic analysis

To analyze the composition of the chemoautotrophic microbial community, the functional genes *cbbL*, *cbbM*, and *accA*, together with the total prokaryotic 16S rRNA gene, were amplified using primer pairs cbbL_K2f/V2r, cbbM-f/r, Crena_529F/981R, and 515f_modified/806r_modified, respectively [29]. Purified amplicons were pooled at equimolar concentrations and sequenced (PE300) on an Illumina MiSeq platform (Illumina, San Diego, USA) following the standard protocols of Majorbio (Shanghai, China). Raw sequences were trimmed, quality-filtered, and assigned to individual samples based on unique barcodes using QIIME [30]. Chimeric sequence detection and removal were performed using the UCHIME algorithm within the USEARCH tool [31]. Subsequently, the sequences were clustered into operational taxonomic units (OTUs) at 95% similarity for functional genes or 97% for 16S rRNA genes using UPARSE [32]. Taxonomic annotation was performed against the NCBI nonredundant database (NRDB) and SILVA’s ribosomal v.138 database using the Ribosomal Database Project (RDP) classifier [33]. Comparative analyses were conducted using a dataset normalized to the lowest sequencing depth across samples. For phylogenetic analysis, the 40 most abundant OTUs and their closest BLAST hits were selected. Maximum-likelihood trees were constructed using IQ-TREE with 1000 ultrafast bootstrap replicates [34], and the resulting trees were visualized using iTOL [35].

### 2.6 Statistical analyses

All statistical analyses were performed using R Statistical Software (v.4.0.2). The normality of variables, including DCF rates, gene abundances, and environmental parameters, was determined using the Shapiro-Wilk test, and the homogeneity of variances was evaluated using a two-tailed F test. One-way ANOVA was used to compare variables among samples. Pearson’s correlation analysis was employed to assess the influence of environmental parameters on DCF and AO rates using the R package linkET [36]. Principal coordinates analysis (PCoA), redundancy analysis (RDA), and canonical correspondence analysis (CCA) were performed to examine the associations between microbial community structure and environmental parameters using the R package vegan [37]. The statistical significance level for all analyses was set to *P* < 0.05.

## 3 Results and discussion

### 3.1 Dark carbon fixation and ammonia oxidation rates

**Fig. 1:**
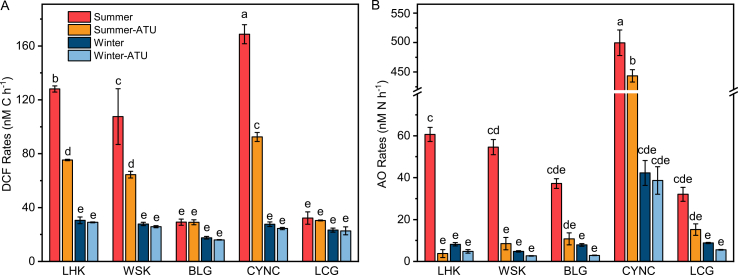
Dark carbon fixation (DCF) rates (A) and ammonia oxidation (AO) rates (B) in water samples from the Yangtze Estuary. Lowercase letters above the bars denote significant differences among samples (*P* < 0.05). Error bars denote standard deviation (*n* = 3).

The DCF process exhibited significantly higher rates in summer (29.18–168.70 nM C/h) than in winter (17.58–30.46 nM C/h; *P* < 0.05), with the highest rate observed at site CYNC (168.70 ± 7.05 nM C/h) and the lowest at site BLG (17.58 ± 0.98 nM C/h; Fig. 1A). These rates were comparable to previous estimates from oxygenated estuarine waters [21,38], but were significantly lower than those reported for chemoclines and suboxic water masses [20]. In summer, the addition of ATU significantly reduced DCF rates at all sites except BLG and LCG (*P* < 0.05), with inhibition ranging from 40% to 45% (Fig. 1A). This is consistent with observations in surface seawater of the Eastern South Pacific, where ATU inhibited more than 30% of DCF activity [39]. In contrast, ATU caused only minor reductions in DCF rates during winter. This seasonal difference may indicate a greater relative contribution of AOA in winter estuarine waters, because ATU has limited effects on AOA activity at the concentration used in this study [40].

The AO rates were significantly higher in summer (32.05–499.63 nM N/h) than in winter (4.78–42.24 nM N/h; *P* < 0.05), with peak rates observed at site CYNC in both seasons (Fig. 1B). Our AO rates were comparable to those reported in nearshore water of the Yangtze Estuary (0–191.7 nM N/h), Pearl River Estuary (108.2–251.7 nM N/h), and Schelde Estuary (4.0–701.5 nM N/h), but were significantly higher than those reported for offshore waters of the Yangtze Estuary (0–20.8 nM N/h) [41,42]. This nearshore-offshore disparity is likely associated with the greater availability of NH_4_^+^ in estuarine environments [43]. ATU addition reduced AO rates by 10% at site CYNC but resulted in significant decreases (37%–94%; *P* < 0.05) at other sites. These inhibition ratios were substantially higher than those observed (10%–20%) in the Eastern Tropical North Pacific, suggesting that AOB may contribute more substantially to AO process in estuaries than in open-ocean waters [44]. This difference likely arises from the lower salt tolerance and reduced transcriptional activity of AOB under high-salinity environments [45,46].

### 3.2 Variation of environmental properties

**Fig. 2:**
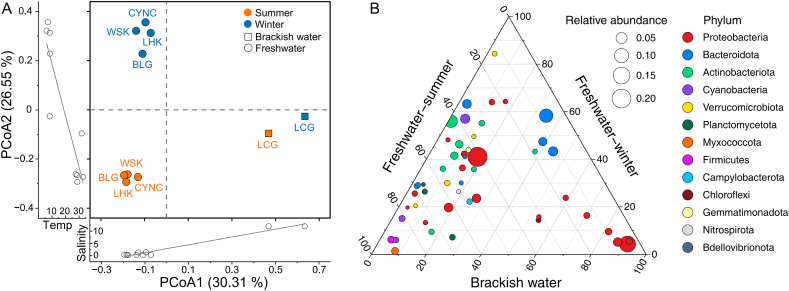
Environmental drivers of microbial community composition in waters of the Yangtze Estuary. (A) Principal coordinate analysis (PCoA) based on Bray-Curtis dissimilarity of bacterial and archaeal 16S rRNA sequences. The bottom panel shows the correlation between the first principal coordinate and salinity, and the left panel shows the correlation between the second principal coordinate and temperature. (B) Ternary plot depicting the relative occurrence of individual orders affiliated with the most abundant phyla in the water samples. Orders are colored by their phylum, and circle size is proportional to their mean abundance in the community.

Salinity is a primary hydrographic parameter in estuarine ecosystems because it reflects the interplay between riverine and marine waters. In this study, salinity ranged from 0.18 to 0.26 psu at upstream sites, increased to 1.36 psu at site CYNC, and reached to 11.96 psu at site LCG (Table S2). The mean water temperature during the summer surveys was 22.2 °C higher than during the winter surveys. The combined effects of salinity and temperature drove the clustering of microbial communities in estuarine water into three distinct groups: summer freshwater group, winter freshwater group, and brackish water group (Fig. 2A). These results indicate that both seasonal temperature variation and spatial salinity gradients shaped prokaryotic community structure in the estuarine waters [47,48]. The prokaryotic community was dominated by Proteobacteria (49.7%), Bacteroidota (17.1%), and Actinobacteriota (13.4%) (Fig. 2B). At the order level, Burkholderiales were enriched in freshwater sites (22.2%–30.6%), whereas Rhodobacterales were significantly more abundant in brackish waters (23.3%–37.9%) than in freshwater sites (0.9%–3.7%; *P* < 0.05) (Fig. S3).

NH_4_^+^ concentration was exceptionally high at site CYNC in summer (972.27 ± 46.01 μM; *P* < 0.001; Table S2), probably owing to the proximity of this site to landfill inputs [49]. The concentrations of NO_2_^−^, SiO_3_^2−^, and PO_4_^3−^ ranged from 0.89 to 22.13 μM, 59.05–124.63 μM, and 0.01–5.38 μM, respectively, exhibiting significant spatial heterogeneity (*P* < 0.05). During the investigation, a decrease in DO was observed in summer water samples at sites LHK, WSK, and BLG, although DO levels remained within the oxic range (4.4–6.9 mg/L).

### 3.3 Dynamics of chemoautotrophic communities using the CBB and 3-HP/4-HB pathways

**Fig. 3:**
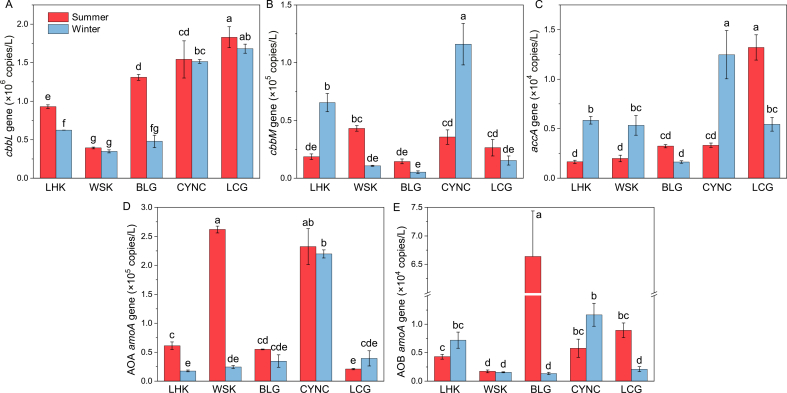
Abundances of *cbbL* (A), *cbbM* (B), *accA* (C), archaeal *amoA* (D), and bacterial *amoA* (E) genes in water samples from the Yangtze Estuary. Lowercase letters above the bars denote significant differences among samples (*P* < 0.05). Error bars denote standard deviation (*n* = 3).

The qPCR technique was employed to quantify the abundance of *cbbL*, *cbbM*, and *accA* genes, as well as the archaeal and bacterial *amoA* genes. The abundance of the *cbbL* gene ranged from 3.48 × 10^5^ to 1.83 × 10^6^ copies/L, with significantly higher values at sites CYNC and LCG than at other sites (*P* < 0.05; Fig. 3A). These values were comparable to those reported for non-estuarine turbidity maxima (non-ETM) waters of the Columbia River estuary, but lower than those observed in ETM waters [38]. The abundance of the *cbbM* gene was an order of magnitude lower than that of the *cbbL* gene (Fig. 3B), with peak abundance detected at site CYNC (1.16 × 10^5^ copies/L) and the lowest at site BLG (5.35 × 10^3^ copies/L). The abundance of *accA* gene ranged from 1.63 × 10^3^ to 1.32 × 10^4^ copies/L and exhibited a discernible increasing trend with salinity (Fig. 3C). In contrast, Archaeal and bacterial *amoA* abundances showed no significant seasonal or spatial variation, ranging from 0.17 × 10^5^ to 2.62 × 10^5^ copies/L for AOA and from 0.13 × 10^4^ – 6.63 × 10^4^ copies/L for AOB, respectively (Fig. 3D and E).

**Fig. 4:**
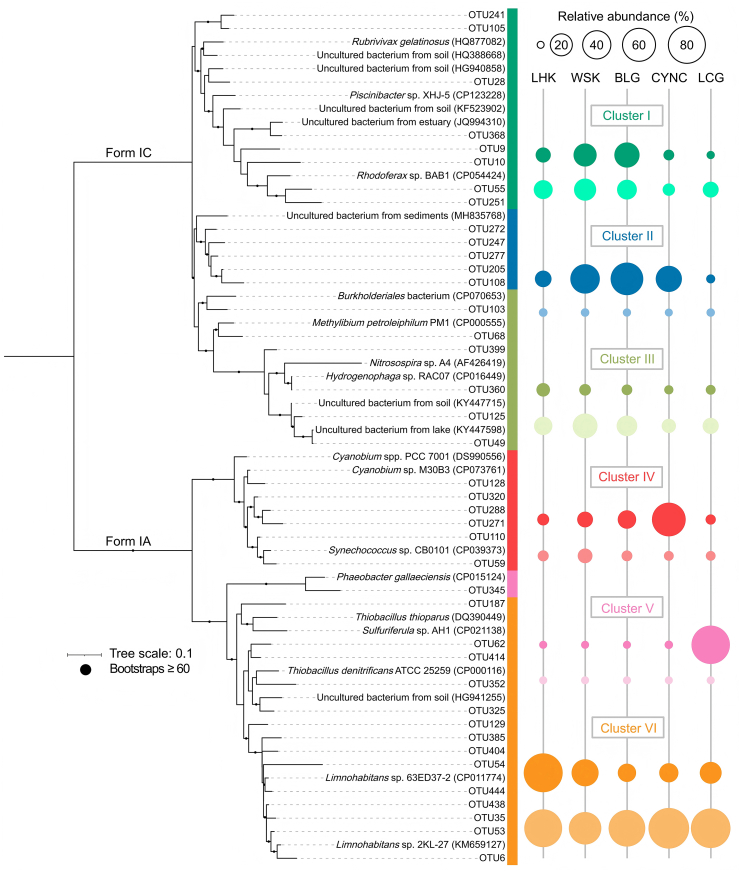
Maximum-likelihood tree of the 40 most abundant representative *cbbL* OTU sequences and their closest relatives from the NCBI database, constructed using IQ-TREE with the GTR + F + I + G4 substitution model. Branch lengths represent base substitutions per sequence site. Nodes with bootstrap values ≥ 60% are marked with black circles. Different sectors are color-coded according to the phylogenetic affiliation of the clusters. The right panel shows the relative abundances of *cbbL* OTUs across samples: summer samples are denoted by high saturation circles, and winter samples by low saturation circles within each cluster.

Amplicon sequencing of *cbbL*, *cbbM*, and *accA* genes was used to link chemoautotrophs containing RubisCO and ACCase with known phylogenetic lineages. The 40 most abundant *cbbL* OTUs accounted for 81.6% of all *cbbL* reads, whereas only 14 of the 451 *cbbL* OTUs were shared across all samples. Phylogenetic analysis of the *cbbL* sequences showed that Form IA and Form IC RubisCO were retrieved (Fig. 4). Taxonomically, most of OTUs were closely related to Burkholderiales, with distinct RubisCO forms displaying different distribution patterns across the estuary. Most OTUs in the Form IA branch (cluster VI) shared high sequence similarity (85.4%–97.8%) with *Limnohabitans* sp. (CP011774 and KM659127), a lineage previously detected in association with *Nitrosomonas* and *Nitrosopumilus* in AOB consortia and potentially involved in AO-related carbon fixation [50,51]. The relative abundance of cluster VI OTUs was significantly higher in winter (53.2%–94.4%) than in summer (9.5%–84.6%; *P* < 0.05). Within the Form IA branch, one OTU in cluster V shared 88.1% sequence identity with *Phaeobacter gallaeciensis* (CP015124), and six OTUs in cluster IV shared 92.6%–95.1% sequence identity with *Cyanobium* sp. (CP073761). These Alphaproteobacteria- and Cyanobacteria-associated OTUs were less dominant than Burkholderiales-associated OTUs, consistent with previous evidence that AOB-related chemoautotrophs can be important contributors to DCF in oxygenated waters [8]. In addition, several other AOB strains can fix carbon utilizing Form IC RubisCO. These OTUs within the Form IC branch shared 74.3%–85.1% sequence identity with *Nitrospira* sp. (AF426419) (Fig. 4). However, numerous OTUs within the Form IC branch were not explicitly linked to known chemoautotrophic strains, indicating gaps in our understanding of this functional group. Although AOB with Form IA RubisCO exhibit low CO_2_ affinity and tolerance to high NH_4_^+^ and NO_2_^−^ concentrations, they can thrive in low-CO_2_ environments through CO_2_-concentrating mechanisms [52,53]. In contrast, AOB with Form IC RubisCO exhibit higher CO_2_ affinity, indicating their adaptation to environments with high CO_2_ and O_2_ levels [52]. Several AOB species, including *Nitrosomonas oligotropha* N. Is79 and N. JL21, possess both Form IA and IC RubisCOs, indicating their niche adaptation and diverse strategies to cope with fluctuating CO_2_, O_2_, and NH_4_^+^ levels [53,54]. This diversity enables AOB communities to effectively adapt to the dynamic environmental conditions of estuaries.

For *cbbM* gene, which encodes Form II RubisCO, a total of 643 OTUs were generated using a distance cutoff of 0.05. The 40 most abundant *cbbM* OTUs accounted for 78.2% of all *cbbM* reads, with pairwise sequence similarities ranging from 64.3% to 95.0%. The maximum-likelihood tree separated these OTUs into six clusters with distinct distribution patterns (Fig. S4). Clusters I and V exhibited similar seasonal variation in their ecological niche distributions, with higher relative abundances in summer (49.7% and 29.7%, respectively) than in winter (0.2% and 1.0%, respectively). However, their responses to salinity differed: the relative abundance of cluster I increased with salinity, whereas cluster V predominated in freshwater. These patterns indicate that while microorganisms harboring the *cbbM* gene were widespread across salinity gradients, their community composition and structure were strongly influenced by salinity [37,55]. Cluster I OTUs were closely related to Burkholderiales, whereas cluster IV OTUs could not be assigned to known chemoautotrophic taxa, indicating that our understanding of this freshwater chemoautotrophic community remains limited. The relative abundance of cluster II OTUs was significantly lower in summer (18.5%) than in winter (54.8%). The most abundant *cbbM* OTU, OTU620 in cluster II, accounted for 17.8% of reads and shared 96.9% identity with *Limnohabitans* sp. (KM659124; Fig. S4). *Limnohabitans* sp., like other chemoautotrophs such as *Thiobacillus denitrificans* and *Sulfuricella denitrificans*, is known to possess both Form I and II RubisCO [37,52]. Compared to Form I RubisCO, Form II enzymes have lower affinity for CO_2_ and reduced ability to discriminate against O_2_ as a substrate [52], adapting them to environments with high CO_2_ and low O_2_ [8,37]. The co-occurrence of Form I and II RubisCOs in chemoautotrophs thus represents an evolutionary strategy to cope with frequent fluctuations in O_2_ and CO_2_ concentrations in dynamic estuarine environments.

Phylogenetic analysis of the *accA* sequences identified four major clusters (Fig. S5). Cluster I, consisting of 12 OTUs, was closely related to the family Nitrosopumilaceae and shared 80.6%–96.1% sequence similarity with *Nitrosopumilus maritimus* SCM1 (CP000866). This widely distributed archaeon, common in oceanic environments, is known for its high affinity for NH_4_^+^ [15]. *N. maritimus* utilizes the 3-HP/4-HB cycle for CO_2_ assimilation, contributing significantly to carbon and nitrogen biogeochemical cycles due to its broad environmental distribution [17,56,57]. Cluster I OTUs accounted for 77.1% of *accA* sequences at sites BLG and LCG, but only for 4.9% at the other sites, likely reflecting the preference of many AOA lineages for moderate-to high-salinity environments [10,45,58]. Cluster II OTUs shared 86.8%–91.9% sequence similarity with *Candidatus Nitrosotenuis cloacae* (CP011097; Fig. S5), an AOA strain isolated from freshwater [59]. Because the “*Ca. Nitrosotenuis*” clade lacks complete pathways for compatible-solute synthesis, it is sensitive to elevated salinity [59,60]. Consequently, this lineage was detected only at the three upstream sites, with an average relative abundance of 9.2%. These results suggest that niche differentiation within AOA communities facilitates adaptation to dynamic fluctuations in salinity and NH_4_^+^ levels in estuarine environments [61].

### 3.4 Effects of biotic and abiotic factors on the DCF process and its ecological significance

**Fig. 5:**
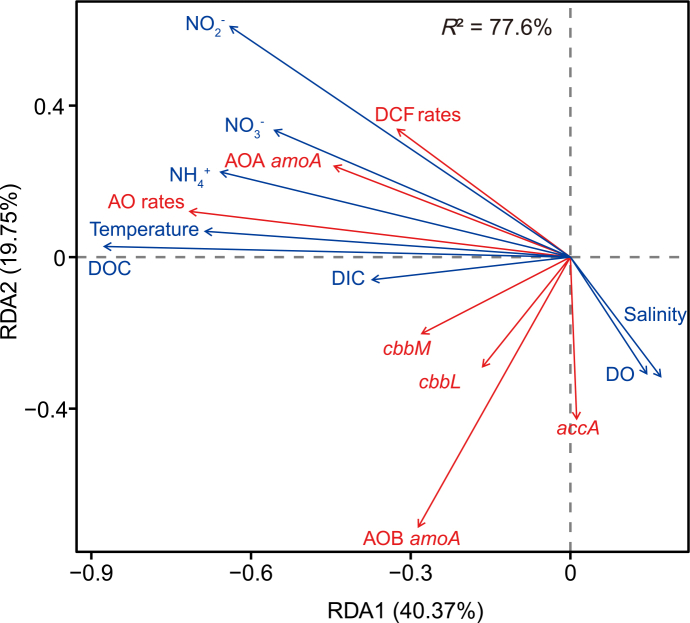
Redundancy analysis (RDA) of DCF and AO rates, as well as functional gene abundances, constrained by physicochemical parameters in water samples from the Yangtze Estuary. The numbers on each axis represent the percentage of total variance explained.

**Fig. 6:**
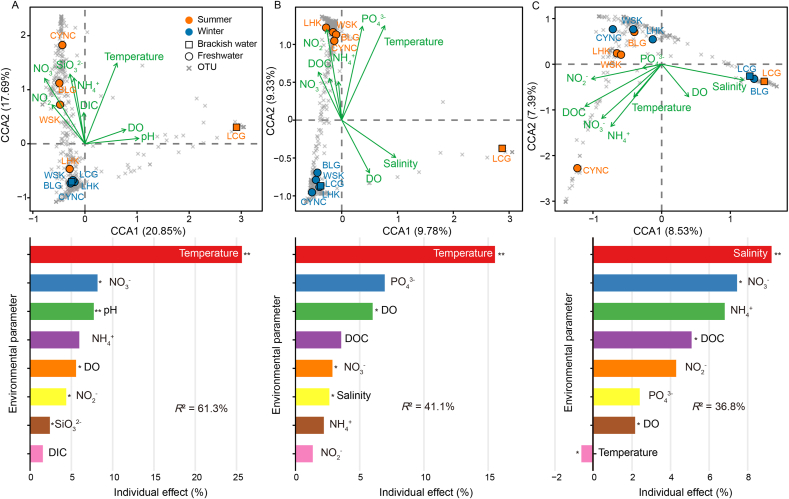
Canonical correspondence analysis (CCA) showing factors influencing *cbbL*- (A), *cbbM*- (B), and *accA*-harboring (C) chemoautotrophic communities. The numbers on each axis represent the percentage of total variance explained. Individual impacts of environmental parameters are shown in the bottom panel. ∗*P* < 0.05; ∗∗*P* < 0.01; ∗∗∗*P* < 0.001.

In this study, we analyzed the potential effects of biotic and abiotic factors on the microbial-mediated DCF process. DCF rates in the Yangtze estuarine waters were 28%–83% higher in summer than in winter. RDA and Pearson’s correlation analyses revealed a positive correlation between DCF rates and temperature (*R* = 0.67, *P* < 0.05; Fig. 5 and S6). This seasonal variation likely reflects the temperature dependence of microbial enzymatic reactions [27], although different microbial groups may vary in their physiological and kinetic responses to temperature [62]. ATU-amended incubations indicated that AOB-associated chemoautotrophy contributed an average of 42.1% to the DCF process at sites LHK, WSK, and CYNC in summer. Temperature was also a major environmental factor associated with the composition of CBB-associated chemoautotrophs, explaining 25.3% and 15.3% of the total variation in *cbbL*- and *cbbM*-harboring communities, respectively (Fig. 6). In contrast, AOA generally have broader thermal tolerance and lower temperature sensitivity in growth responses [63], suggesting that they may play a significant role in the DCF process during winter [64]. Salinity was the dominant factor shaping chemoautotrophic microbial community harboring the 3-HP/4-HB cycle (Fig. 6C), as reflected by the contrasting distributions of freshwater-associated *Ca. Nitrosotenuis* and brackish-water-associated *N. maritimus* lineages (Fig. S5). Notably, the abundances of marker genes were not significantly correlated with DCF rates (*P* > 0.05; Fig. S6), indicating that gene abundance alone may not reliably predict the activity or contribution of different chemoautotrophic groups [12]. Future direct measurements of the actual activity of these chemoautotrophs will likely enhance our understanding of their biogeochemical significance.

Nitrification provides an important energy source for chemoautotrophic carbon fixation in oxygenated waters, particularly through microorganisms using the CBB and 3-HP/4-HB pathways [8,13]. In this study, Pearson’s correlation and RDA analyses revealed a significantly positive correlation (*R* = 0.78, *P* < 0.01) between DCF rates and AO rates (Figs. 5 and S6), consistent with observations from the Baltic Sea [13]. DCF and AO rates were also positively correlated with NH_4_^+^ (*R* = 0.70 and 0.99, respectively), DOC (*R* = 0.66 and 0.87, respectively), and AOA *amoA* gene abundance (*R* = 0.59 and 0.55, respectively). These relationships indicate that NH_4_^+^ availability is a major driver of both DCF and AO processes [65]. Previous studies have also demonstrated that nitrifiers benefit from the NH_4_^+^ generated through the heterotrophic bacterial remineralization of organic matter [65,66].

There is a growing consensus that DCF represents an integral component of marine food webs and should be considered in assessments of marine primary productivity [6]. Based on the measured rates, chemoautotrophic carbon fixation accounted for 2.9%–16.8% of phytoplankton primary production in coastal waters adjacent to the Yangtze Estuary during summer, increasing to 33.8%–58.6% during winter [67]. These proportions are lower than those reported for suboxic and anoxic waters of the Black Sea (45%–83%) [68], but they nevertheless indicate that DCF can represent a substantial fraction of microbial carbon production in estuarine and coastal waters. Similarly, Piontek et al. [65] reported that DCF in sulfide-free water accounted for 9%–54% of heterotrophic microbial carbon demand in the Baltic Sea. Together, these results highlighted that chemoautotrophic DCF contributes meaningfully to carbon cycling in blue carbon ecosystems, and its ecological significance should not be overlooked in coastal carbon assessments.

## 4 Conclusion

This study combined ^14^C and ^15^N isotope probing techniques to simultaneously investigate the rates of DCF and AO processes in estuarine waters. Significant positive correlations were found among DCF rates, AO rates, and NH_4_^+^ levels, indicating that nitrifiers played a crucial role in the DCF and AO processes in estuarine environments. Temperature was identified as a key factor shaping the community structure of CBB-associated chemoautotrophs, which were mainly affiliated with Burkholderiales, whereas salinity and NH_4_^+^ levels significantly influenced the composition of 3-HP/4-HB-associated chemoautotrophic communities, which were dominated by Nitrosopumilaceae. Niche differentiation within nitrifier communities enables them to effectively adapt to frequent environmental fluctuations (including temperature, salinity, and NH_4_^+^ concentration) in estuarine regions, thereby contributing significantly to nitrogen cycling and carbon sequestration in these ecosystems. These findings highlight the ecological significance of estuarine nitrifiers within the food webs and emphasize the need to incorporate the DCF process into the blue carbon framework for estuarine and coastal ecosystems. Future research should not only assess the activity of chemoautotrophs at the transcriptional and translational levels but also trace the fate of chemoautotrophic organic carbon, thereby providing further insights into their biogeochemical significance.

## CRediT authorship contribution statement

**Bolin Liu:** Conceptualization, Formal analysis, Funding acquisition, Investigation, Visualization, Writing – original draft. **Xinyu Wang:** Investigation. **Can Jiang:** Visualization. **Lin Qi:** Methodology. **Qianqian Bi:** Methodology. **Guoyu Yin:** Formal analysis. **Hongpo Dong:** Formal analysis. **Xiaofei Li:** Formal analysis. **Xia Liang:** Formal analysis. **Ping Han:** Formal analysis. **Min Liu:** Funding acquisition. **Yanling Zheng:** Conceptualization, Funding acquisition, Supervision, Writing – review & editing. **Lijun Hou:** Conceptualization, Funding acquisition, Supervision, Writing – review & editing.

## Declaration of competing interest

The authors declare that they have no known competing financial interests or personal relationships that could have appeared to influence the work reported in this paper.

## References

[1] Griscom B.W., Adams J., Ellis P.W., Houghton R.A., Lomax G., Miteva D.A. (2017). Natural climate solutions. Proc. Natl. Acad. Sci. USA.

[2] Bauer J.E., Cai W.-J., Raymond P.A., Bianchi T.S., Hopkinson C.S., Regnier P.A.G. (2013). The changing carbon cycle of the coastal ocean. Nature.

[3] Macreadie P.I., Costa M.D.P., Atwood T.B., Friess D.A., Kelleway J.J., Kennedy H. (2021). Blue carbon as a natural climate solution. Nat. Rev. Earth Environ..

[4] Middelburg J.J. (2011). Chemoautotrophy in the ocean. Geophys. Res. Lett..

[5] Saxena H., Sahoo D., Nazirahmed S., Rai D.K., Khan M.A., Sharma N. (2022). Contribution of carbon fixation toward carbon sink in the ocean twilight zone. Geophys. Res. Lett..

[6] Baltar F., Herndl G.J. (2019). Ideas and perspectives: is dark carbon fixation relevant for oceanic primary production estimates?. Biogeosciences.

[7] Yu X., Qian L., Tu Q., Peng Y., Wang C., Wu D. (2023). Chemoautotrophic sulphur oxidizers dominate microbial necromass carbon formation in coastal blue carbon ecosystems. Funct. Ecol..

[8] Alfreider A., Baumer A., Bogensperger T., Posch T., Salcher M.M., Summerer M. (2017). CO_2_ assimilation strategies in stratified lakes: diversity and distribution patterns of chemolithoautotrophs. Environ. Microbiol..

[9] Wu J., Hong Y., He X., Liu X., Ye J., Jiao L. (2022). Niche differentiation of ammonia-oxidizing Archaea and related autotrophic carbon fixation potential in the water column of the South China Sea. iScience.

[10] Martens-Habbena W., Berube P.M., Urakawa H., de la Torre J.R., Stahl D.A. (2009). Ammonia oxidation kinetics determine niche separation of nitrifying Archaea and Bacteria. Nature.

[11] Kits K.D., Sedlacek C.J., Lebedeva E.V., Han P., Bulaev A., Pjevac P. (2017). Kinetic analysis of a complete nitrifier reveals an oligotrophic lifestyle. Nature.

[12] Veuger B., Pitcher A., Schouten S., Sinninghe Damsté J.S., Middelburg J.J. (2013). Nitrification and growth of autotrophic nitrifying bacteria and Thaumarchaeota in the coastal North Sea. Biogeosciences.

[13] Berg C., Vandieken V., Thamdrup B., Jürgens K. (2015). Significance of archaeal nitrification in hypoxic waters of the Baltic Sea. ISME J..

[14] Daims H., Lebedeva E.V., Pjevac P., Han P., Herbold C., Albertsen M. (2015). Complete nitrification by *Nitrospira* bacteria. Nature.

[15] Le Coq P., Christaki U., Van Wambeke F., Chevillon E., Zakardjian B., Garel M. (2026). Distinct contributions of suspended and sinking prokaryotes to mesopelagic carbon budget. Nat. Geosci..

[16] Yamamoto A., Hajima T., Yamazaki D., Noguchi Aita M., Ito A., Kawamiya M. (2022). Competing and accelerating effects of anthropogenic nutrient inputs on climate-driven changes in ocean carbon and oxygen cycles. Sci. Adv..

[17] Damashek J., Francis C.A. (2018). Microbial nitrogen cycling in estuaries: from genes to ecosystem processes. Estuaries Coasts.

[18] Sun D., Tang X., Zhao M., Zhang Z., Hou L., Liu M. (2020). Distribution and diversity of comammox *Nitrospira* in coastal wetlands of China. Front. Microbiol..

[19] Zhou J., Zheng Y., Hou L., An Z., Chen F., Liu B. (2023). Effects of acidification on nitrification and associated nitrous oxide emission in estuarine and coastal waters. Nat. Commun..

[20] Lee D.Y., Owens M.S., Crump B.C., Cornwell J.C. (2015). Elevated microbial CO_2_ production and fixation in the oxic/anoxic interface of estuarine water columns during seasonal *Anoxia*. Estuar. Coast Shelf Sci..

[21] Signori C.N., Valentin J.L., Pollery R.C.G., Enrich-Prast A. (2018). Temporal variability of dark carbon fixation and bacterial production and their relation with environmental factors in a tropical estuarine system. Estuaries Coasts.

[22] Tan Q., Zhu Y., Zhao Y., Zheng L., Wang X., Xing Y. (2025). Comparative analysis of niche adaptation strategies of AOA, AOB, and comammox along a gate-controlled river-estuary continuum. Water Res..

[23] Wu Y., Zhang J., Liu S.M., Zhang Z.F., Yao Q.Z., Hong G.H. (2007). Sources and distribution of carbon within the Yangtze River system. Estuar. Coast Shelf Sci..

[24] Tong Y., Zhao Y., Zhen G., Chi J., Liu X., Lu Y. (2015). Nutrient loads flowing into coastal waters from the main rivers of China (2006-2012). Sci. Rep..

[25] Findlay S., McDowell W.H., Fischer D., Pace M.L., Caraco N., Kaushal S.S. (2010). Total carbon analysis may overestimate organic carbon content of fresh waters in the presence of high dissolved inorganic carbon. Limnol Oceanogr. Methods.

[26] Alonso-Sáez L., Galand P.E., Casamayor E.O., Pedrós-Alió C., Bertilsson S. (2010). High bicarbonate assimilation in the dark by Arctic bacteria. ISME J..

[27] Qi L., Zheng Y., Hou L., Liu B., Zhou J., An Z. (2023). Potential response of dark carbon fixation to global warming in estuarine and coastal waters. Glob. Change Biol..

[28] Martens-Habbena W., Qin W., Horak R.E., Urakawa H., Schauer A.J., Moffett J.W. (2015). The production of nitric oxide by marine ammonia-oxidizing Archaea and inhibition of archaeal ammonia oxidation by a nitric oxide scavenger. Environ. Microbiol..

[29] Walters W., Hyde E.R., Berg-Lyons D., Ackermann G., Humphrey G., Parada A. (2016). Improved bacterial 16S rRNA gene (V4 and V4-5) and fungal internal transcribed spacer marker gene primers for microbial community surveys. mSystems.

[30] Caporaso J.G., Kuczynski J., Stombaugh J., Bittinger K., Bushman F.D., Costello E.K. (2010). QIIME allows analysis of high-throughput community sequencing data. Nat. Methods.

[31] Edgar R.C., Haas B.J., Clemente J.C., Quince C., Knight R. (2011). UCHIME improves sensitivity and speed of *Chimera* detection. Bioinform. Oxf. Engl..

[32] Edgar R.C. (2013). UPARSE: highly accurate OTU sequences from microbial amplicon reads. Nat. Methods.

[33] Cole J.R., Wang Q., Fish J.A., Chai B., McGarrell D.M., Sun Y. (2014). Ribosomal Database Project: data and tools for high throughput rRNA analysis. Nucleic Acids Res..

[34] Nguyen L.T., Schmidt H.A., von Haeseler A., Minh B.Q. (2015). IQ-TREE: a fast and effective stochastic algorithm for estimating maximum-likelihood phylogenies. Mol. Biol. Evol..

[35] Letunic I., Bork P. (2019). Interactive Tree of Life (iTOL) v4: recent updates and new developments. Nucleic Acids Res..

[36] Huang H.Y. (2021). https://github.com/Hy4m/linkET.

[37] Liu B., Hou L., Zheng Y., Zhang Z., Tang X., Mao T. (2022). Dark carbon fixation in intertidal sediments: controlling factors and driving microorganisms. Water Res..

[38] Bräuer S.L., Kranzler K., Goodson N., Murphy D., Simon H.M., Baptista A.M. (2013). Dark carbon fixation in the Columbia River’s estuarine turbidity maxima: molecular characterization of red-type *cbbL* genes and measurement of DIC uptake rates in response to added electron donors. Estuaries Coasts.

[39] Molina V., Farías L. (2009). Aerobic ammonium oxidation in the oxycline and oxygen minimum zone of the eastern tropical South Pacific off northern Chile (∼20°S). Deep Sea Res. Part II Top. Stud. Oceanogr..

[40] Yakimov M.M., Cono V.L., Smedile F., DeLuca T.H., Juárez S., Ciordia S. (2011). Contribution of crenarchaeal autotrophic ammonia oxidizers to the dark primary production in Tyrrhenian deep waters (Central Mediterranean Sea). ISME J..

[41] Hsiao S.S.Y., Hsu T.-C., Liu J.-W., Xie X., Zhang Y., Lin J. (2014). Nitrification and its oxygen consumption along the turbid Chang Jiang River plume. Biogeosciences.

[42] Zou D., Li Y., Kao S.J., Liu H., Li M. (2019). Genomic adaptation to eutrophication of ammonia-oxidizing Archaea in the Pearl River estuary. Environ. Microbiol..

[43] Zheng Z.-Z., Zheng L.-W., Xu M.N., Tan E., Hutchins D.A., Deng W. (2020). Substrate regulation leads to differential responses of microbial ammonia-oxidizing communities to ocean warming. Nat. Commun..

[44] Peng X., Fuchsman C.A., Jayakumar A., Oleynik S., Martens-Habbena W., Devol A.H. (2015). Ammonia and nitrite oxidation in the eastern tropical north Pacific. Glob. Biogeochem. Cycles.

[45] Fukushima T., Wu Y.J., Whang L.M. (2012). The influence of salinity and ammonium levels on *AmoA* mRNA expression of ammonia-oxidizing prokaryotes. Water Sci. Technol..

[46] Bernhard A.E., Donn T., Giblin A.E., Stahl D.A. (2005). Loss of diversity of ammonia-oxidizing bacteria correlates with increasing salinity in an estuary system. Environ. Microbiol..

[47] Lozupone C.A., Knight R. (2007). Global patterns in bacterial diversity. Proc. Natl. Acad. Sci. USA.

[48] Righetti D., Vogt M., Gruber N., Psomas A., Zimmermann N.E. (2019). Global pattern of phytoplankton diversity driven by temperature and environmental variability. Sci. Adv..

[49] Song L., Wang Y., Zhao H., Long D.T. (2015). Composition of bacterial and archaeal communities during landfill refuse decomposition processes. Microbiol. Res..

[50] Kasalický V., Jezbera J., Hahn M.W., Šimek K. (2013). The diversity of the *Limnohabitans* genus, an important group of freshwater bacterioplankton, by characterization of 35 isolated strains. PLoS One.

[51] Baskaran V., Patil P.K., Antony M.L., Avunje S., Nagaraju V.T., Ghate S.D. (2020). Microbial community profiling of ammonia and nitrite oxidizing bacterial enrichments from brackishwater ecosystems for mitigating nitrogen species. Sci. Rep..

[52] Badger M.R., Bek E.J. (2008). Multiple Rubisco forms in proteobacteria: their functional significance in relation to CO_2_ acquisition by the CBB cycle. J. Exp. Bot..

[53] Sedlacek C.J., McGowan B., Suwa Y., Sayavedra-Soto L., Laanbroek H.J., Stein L.Y. (2019). A physiological and genomic comparison of *Nitrosomonas cluster* 6a and 7 ammonia-oxidizing bacteria. Microb. Ecol..

[54] Bollmann A., Sedlacek C.J., Norton J., Laanbroek H.J., Suwa Y., Stein L.Y. (2013). Complete genome sequence of *Nitrosomonas* sp. Is79, an ammonia oxidizing bacterium adapted to low ammonium concentrations. Stand. Genom. Sci..

[55] Wang B., Huang J., Yang J., Jiang H., Xiao H., Han J. (2021). Bicarbonate uptake rates and diversity of RuBisCO genes in saline lake sediments. FEMS Microbiol. Ecol..

[56] Walker C.B., de la Torre J.R., Klotz M.G., Urakawa H., Pinel N., Arp D.J. (2010). *Nitrosopumilus maritimus* genome reveals unique mechanisms for nitrification and autotrophy in globally distributed marine crenarchaea. Proc. Natl. Acad. Sci. USA.

[57] Qin W., Zheng Y., Zhao F., Wang Y., Urakawa H., Martens-Habbena W. (2020). Alternative strategies of nutrient acquisition and energy conservation map to the biogeography of marine ammonia-oxidizing Archaea. ISME J..

[58] Zhang Y., Chen L., Dai T., Tian J., Wen D. (2015). The influence of salinity on the abundance, transcriptional activity, and diversity of AOA and AOB in an estuarine sediment: a microcosm study. Appl. Microbiol. Biotechnol..

[59] Li Y., Ding K., Wen X., Zhang B., Shen B., Yang Y. (2016). A novel ammonia-oxidizing archaeon from wastewater treatment plant: its enrichment, physiological and genomic characteristics. Sci. Rep..

[60] Sauder L.A., Engel K., Lo C.C., Chain P., Neufeld J.D. (2018). “Candidatus nitrosotenuis *Aquarius*,” an ammonia-oxidizing archaeon from a freshwater aquarium biofilter. Appl. Environ. Microbiol..

[61] Stuehrenberg J., Kitzinger K., von Arx J.N., Graf J.S., Lavik G., Littmann S. (2025). Urea use drives niche separation between dominant marine ammonia oxidizing Archaea. Nat. Commun..

[62] Alster C.J., Weller Z.D., von Fischer J.C. (2018). A meta-analysis of temperature sensitivity as a microbial trait. Glob. Change Biol..

[63] Duan P., Wu Z., Zhang Q., Fan C., Xiong Z. (2018). Thermodynamic responses of ammonia-oxidizing Archaea and bacteria explain N_2_O production from greenhouse vegetable soils. Soil Biol. Biochem..

[64] Christman G.D., Cottrell M.T., Popp B.N., Gier E., Kirchman D.L. (2011). Abundance, diversity, and activity of ammonia-oxidizing prokaryotes in the coastal Arctic Ocean in summer and winter. Appl. Environ. Microbiol..

[65] Piontek J., Endres S., Le Moigne F.A.C., Schartau M., Engel A. (2019). Relevance of nutrient-limited phytoplankton production and its bacterial remineralization for carbon and oxygen fluxes in the Baltic Sea. Front. Mar. Sci..

[66] Francis C.A., Roberts K.J., Beman J.M., Santoro A.E., Oakley B.B. (2005). Ubiquity and diversity of ammonia-oxidizing Archaea in water columns and sediments of the ocean. Proc. Natl. Acad. Sci. USA.

[67] Zhou W.H., Yuan X.C., Huo W.Y., Yin K.D. (2004). Distribution of chlorophyll a and primary productivity in the adjacent sea area of Changjiang River Estuary. Acta Oceanol. Sin..

[68] Ediger D., Murray J.W., Yilmaz A. (2019). Phytoplankton biomass, primary production and chemoautotrophic production of the Western Black Sea in April 2003. J. Mar. Syst..

