# Supplementary material for: Ecological significance of dark carbon fixation driven by ammonia oxidation in estuarine waters
Source: Eco Environ Health. 2026 Jun 15;5(3):100258. doi: 10.1016/j.eehl.2026.100258 (PMC13382028; doi:10.1016/j.eehl.2026.100258)
Supplement: Multimedia component 1 [file mmc1.docx]

**Ecological significance of dark carbon fixation driven by ammonia oxidation in estuarine waters**

Bolin Liu^a^, Xinyu Wang^a^, Can Jiang^b^, Lin Qi^b^, Qianqian Bi^a^, Guoyu Yin^b^, Hongpo Dong^a^, Xiaofei Li^a^, Xia Liang^a^, Ping Han^c^, Min Liu^b^, Yanling Zheng^b,^*, Lijun Hou^a,^*

^a^State Key Laboratory of Estuarine and Coastal Research, Yangtze Delta Estuarine Wetland Ecosystem Observation and Research Station, East China Normal University, Shanghai 200241, China

^b^Key Laboratory of Geographic Information Science, School of Geographic Sciences, Ministry of Education, East China Normal University, Shanghai 200241, China

^c^School of Energy and Environment and State Key Laboratory of Marine Environmental Health, City University of Hong Kong, Hong Kong SAR, China

*Corresponding authors.

Email: ylzheng@geo.ecnu.edu.cn (Y. Zheng), ljhou@sklec.ecnu.edu.cn (L. Hou)

**Contents of this file**

Supplementary Figures S1–S6

Supplementary Tables S1–S2

# Supplementary Figures


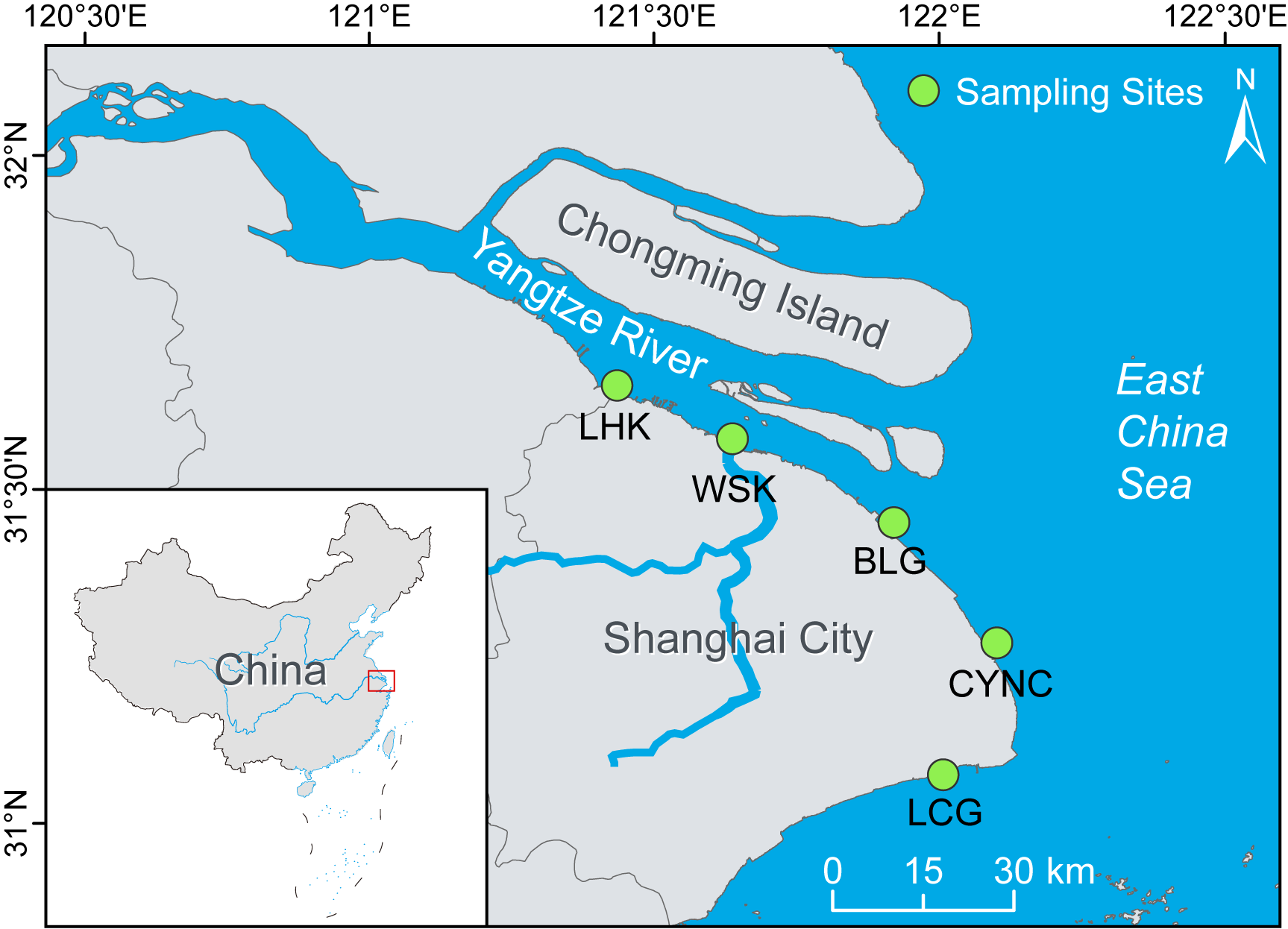


Fig. S1. Map showing the location of the Yangtze Estuary and the sampling sites.


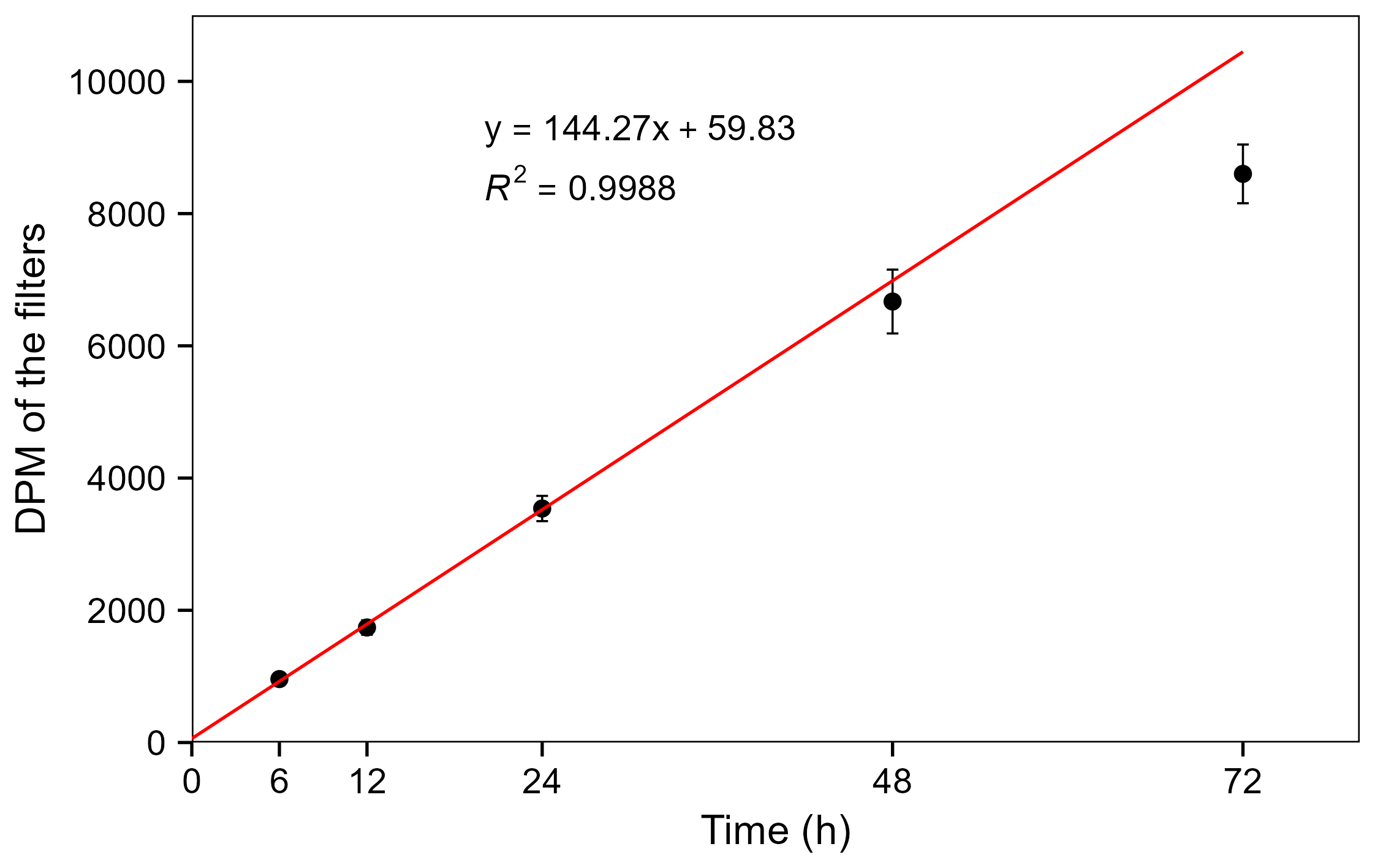


Fig. S2. Time series of the uptake of ^14^C by chemoautotrophs in estuarine waters. DPM (disintegration per minute) denotes the radioactivity of the filters.


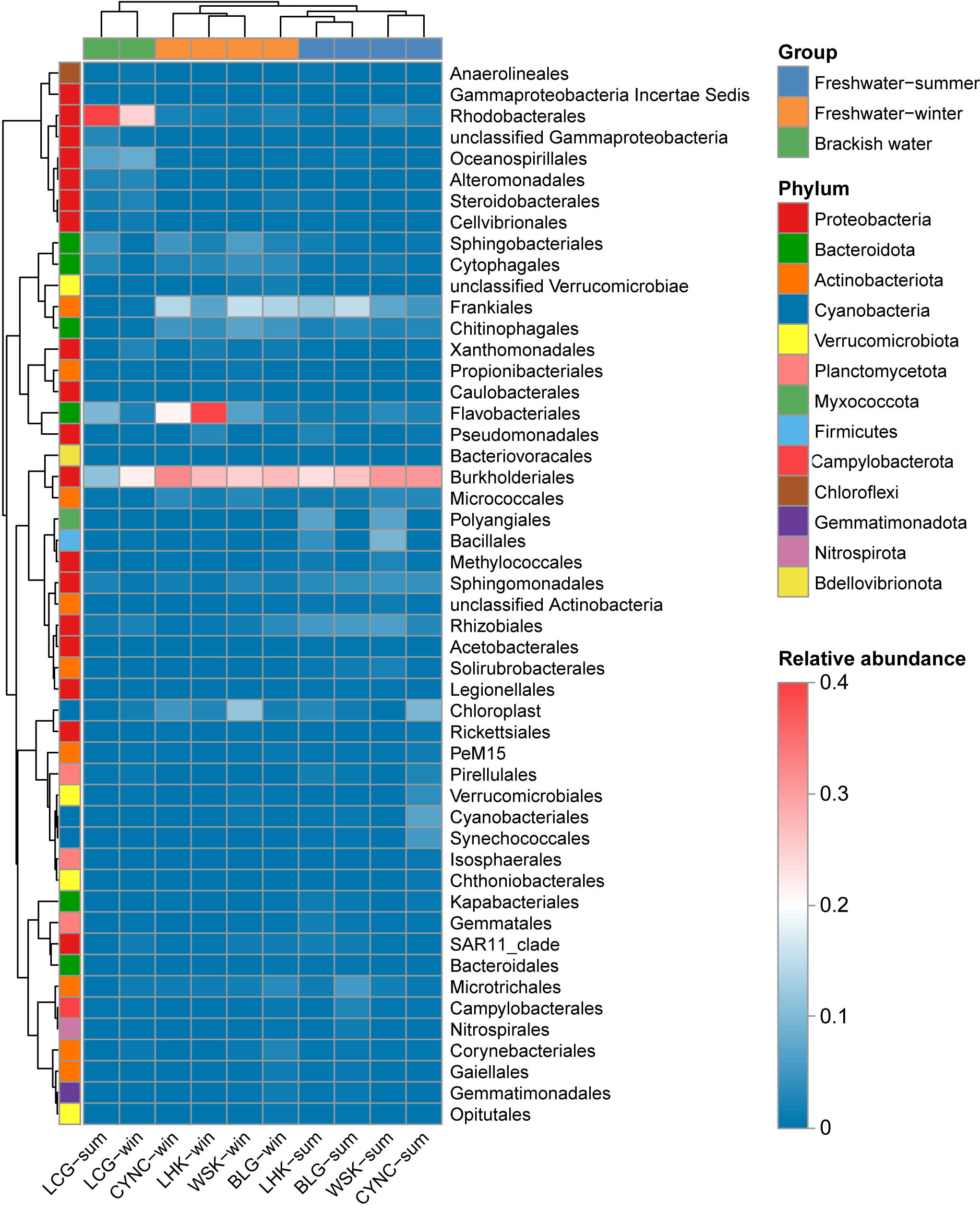


Fig. S3. Heatmap showing the relative abundance of orders within the dominant microbial phyla in Yangtze Estuarine waters. Hierarchical clustering was performed using Pearson correlation (for samples) and Canberra distance (for orders).


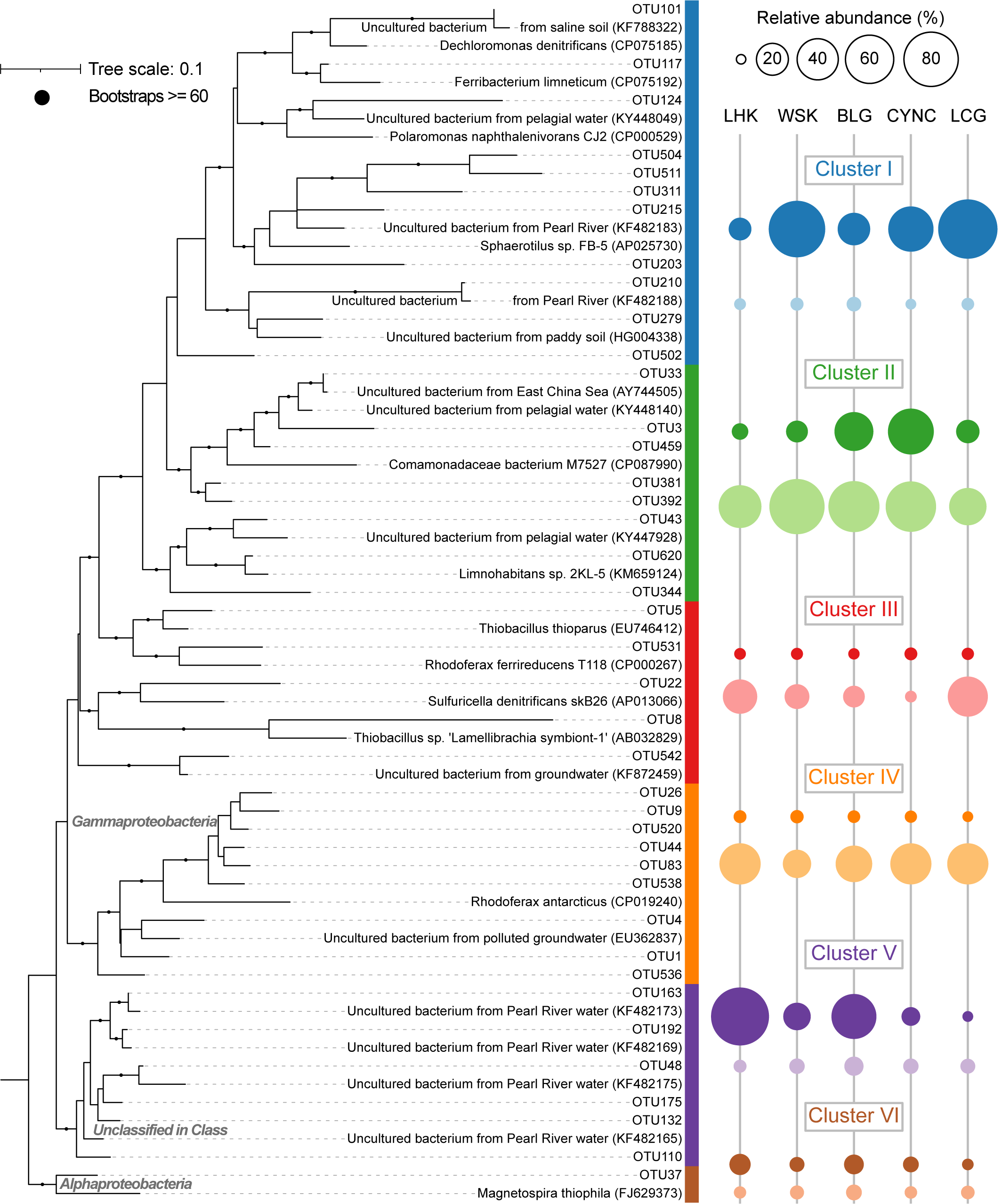


Fig. S4. Maximum-likelihood tree of 40 most abundant representative *cbbM* OTU sequences and their closest relatives from the NCBI database constructed using IQ-TREE with the TIM+F+I+G4 substitution model. Branch lengths represent base substitutions per sequence site. Nodes with bootstrap values ≥ 60% are marked with black circles. Different sectors are color-coded according to the phylogenetic affiliation of the clusters. The right panel shows the relative abundances of *cbbM* genes across samples: summer samples are denoted by high saturation circles, and winter samples by low saturation circles within each cluster.


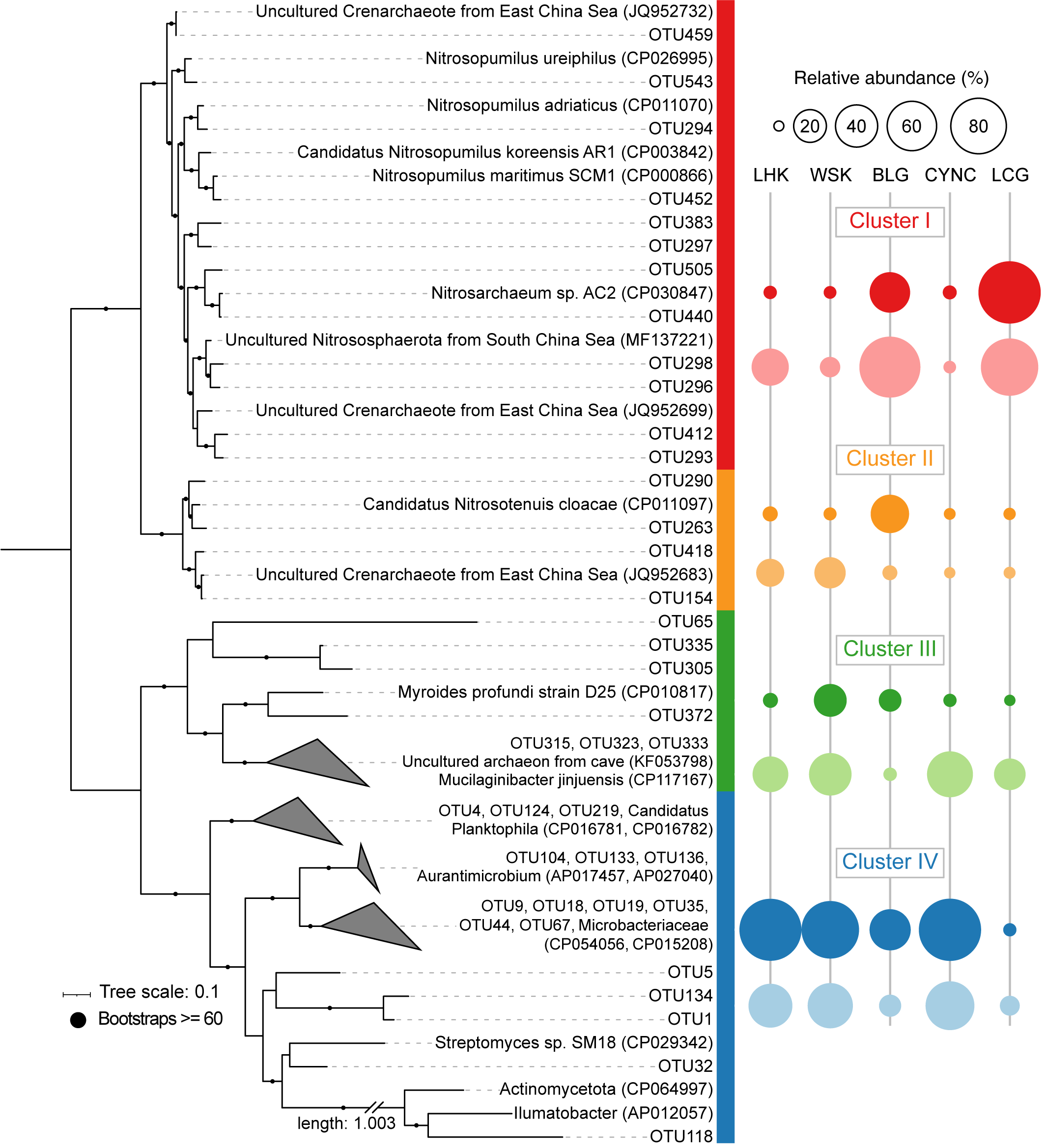


Fig. S5. Maximum-likelihood tree of 40 most abundant representative *accA* OTU sequences and their closest relatives from the NCBI database constructed by IQ-TREE with the SYM+I+G4 substitution model. Branch lengths represent base substitutions per sequence site. Nodes with bootstrap values ≥ 60% are marked with black circles. Different sectors are color-coded according to the phylogenetic affiliation of the clusters. The right panel shows the relative abundances of *accA* genes across samples: summer samples are denoted by high saturation circles, and winter samples by low saturation circles within each cluster.

**
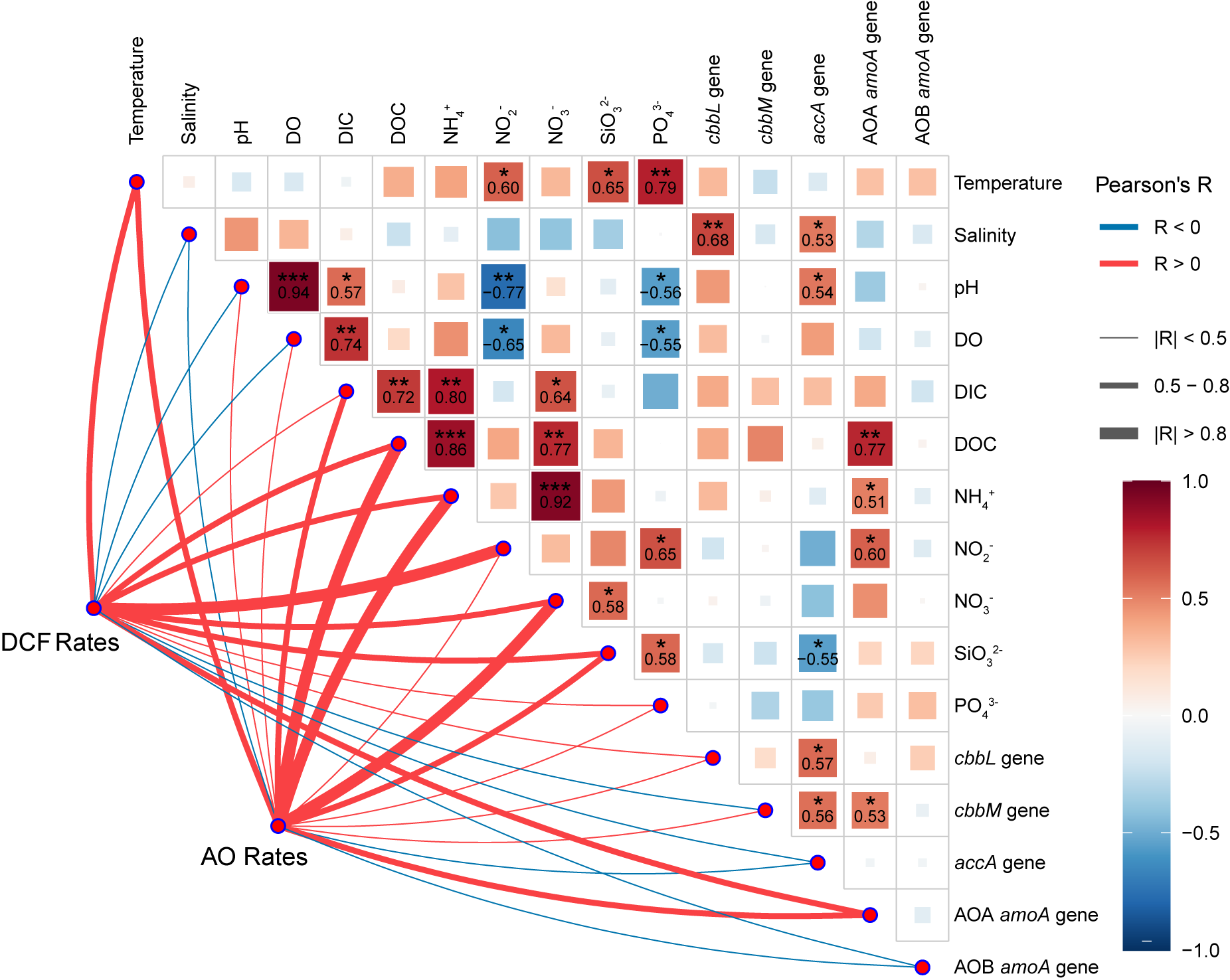
**

Fig. S6. Correlations among dark carbon fixation (DCF) rates, ammonia oxidation (AO) rates, functional gene abundances, and physicochemical characteristics. Values represent Pearson correlation coefficients. Red color represents positive correlation; blue color represents negative correlation; color intensity and square size represent strength of the correlation (**P* < 0.05, ***P* < 0.01, ****P* < 0.001).

# Supplementary Tables

Table S1. Primers and thermal cycling conditions for PCR and qPCR.

| Target gene | Primers | Sequence (5'-3') | Tm | References |
| --- | --- | --- | --- | --- |
| *cbbL* gene | cbbL_K2f  cbbL_V2r | ACCAYCAAGCCSAAGCTSGG  GCCTTCSAGCTTGCCSACCRC | qPCR 63 °C  PCR 62 °C | [1] |
| *cbbM* gene | cbbM-f  cbbM-r | GGCACCATCATCAAGCCCAAG  TCTTGCCGTAGCCCATGGTGC | qPCR 57 °C  PCR 57 °C | [2] |
| *accA* gene | Crena_529F  Crena_981R | GCWATGACWGAYTTTGTYRTAATG  TGGWTKRYTTGCAAYTATWCC | qPCR 50 °C  PCR 48 °C | [3] |
| Archaeal *amoA* gene | Arch-amoAF  Arch-amoAR | STAATGGTCTGGCTTAGACG  GCGGCCATCCATCTGTATGT | qPCR 56 °C | [4] |
| Bacterial *amoA* gene | amoA-1F  amoA-2R | GGGGTTTCTACTGGTGGT  CCCCTCKGSAAAGCCTTCTTC | qPCR 58 °C | [5] |
| Prokaryotic 16S rRNA | 515f_modified  806r_modified | GTGYCAGCMGCCGCGGTAA  GGACTACNVGGGTWTCTAAT | PCR 50 °C | [6] |

Table S2. Physiochemical parameters of the water samples.

| Seasons | Sites | Temp | Salinity | pH | DO | SiO_3_^2–^ | PO_4_^3–^ | NO_2_^–^ | NO_3_^–^ | NH_4_^+^ | DIC | DOC |
| --- | --- | --- | --- | --- | --- | --- | --- | --- | --- | --- | --- | --- |
|  |  | °C | psu |  | mg L^-1^ | μM | μM | μM | μM | μM | μM | μM |
| Summer | LHK | 28.9 | 0.19 | 7.33 | 4.4 | 99.70±4.04 | 3.58±0.04 | 22.13±0.37 | 114.33±0.15 | 22.53±0.31 | 1746.7±4.89 | 244.77±0.88 |
|  | WSK | 27.7 | 0.24 | 7.32 | 5.2 | 114.67±4.22 | 5.38±0.14 | 20.33±0.78 | 121.03±0.62 | 19.40±0.17 | 1905.78±4.34 | 319.43±0.79 |
|  | BLG | 28.6 | 0.26 | 7.79 | 6.9 | 110.17±7.39 | 3.93±0.34 | 5.31±0.05 | 140.87±0.43 | 2.11±0.23 | 1967.97±2.11 | 274.58±1.23 |
|  | CYNC | 33.0 | 1.22 | 8.06 | 10.2 | 124.63±1.97 | 2.05±0.10 | 14.17±0.42 | 323.87±2.45 | 972.27±46.01 | 3488.90±10.1 | 793.39±3.98 |
|  | LCG | 32.0 | 11.90 | 8.23 | 9.7 | 89.87±1.61 | 2.86±0.07 | 1.75±0.01 | 64.87±0.68 | 36.60±0.39 | 2332.09±5.47 | 184.62±0.96 |
| Winter | LHK | 8.6 | 0.18 | 8.03 | 8.6 | 112.80±2.46 | 0.93±0.12 | 2.25±0.02 | 122.20±0.33 | 10.23±0.18 | 2255.15±4.73 | 259.02±0.35 |
|  | WSK | 6.1 | 0.21 | 7.90 | 9.3 | 68.57±2.12 | 0.56±0.23 | 1.99±0.25 | 117.30±13.92 | 9.73±0.74 | 2614.30±2.88 | 121.08±0.52 |
|  | BLG | 8.7 | 0.23 | 7.88 | 7.8 | 94.70±7.66 | 0.79±0.03 | 3.17±0.09 | 152.80±0.15 | 21.25±0.07 | 2055.00±1.86 | 115.24±0.21 |
|  | CYNC | 7.1 | 1.36 | 7.79 | 7.3 | 59.05±6.29 | 0.01±0.00 | 5.90±0.06 | 105.15±0.51 | 82.85±0.96 | 2762.12±5.72 | 515.29±8.90 |
|  | LCG | 8.6 | 11.96 | 7.88 | 8.0 | 71.40±2.66 | 1.66±0.06 | 0.89±0.02 | 86.10±2.37 | 27.30±0.41 | 2362.05±1.27 | 178.21±0.54 |

**References**

[1] K. Nanba, G.M. King, K. Dunfield, Analysis of facultative lithotroph distribution and diversity on volcanic deposits by use of the large subunit of ribulose 1,5-bisphosphate carboxylase/oxygenase, Appl. Environ. Microb. 70 (2004) 2245–2253. https://doi.org/10.1128/AEM.70.4.2245-2253.2004.

[2] A. Alfreider, C. Vogt, D. Hoffmann, W. Babel, Diversity of ribulose-1,5-bisphosphate carboxylase/oxygenase large-subunit genes from groundwater and aquifer microorganisms, Microb. Ecol. 45 (2003) 317–328. https://doi.org/10.1007/s00248-003-2004-9.

[3] M.M. Yakimov, V. La Cono, R. Denaro, A first insight into the occurrence and expression of functional *amoA* and *accA* genes of autotrophic and ammonia-oxidizing bathypelagic Crenarchaeota of Tyrrhenian Sea, Deep-Sea Res. PT. II 56 (2009) 748–754. https://doi.org/10.1016/j.dsr2.2008.07.024.

[4] C.A. Francis, K.J. Roberts, J.M. Beman, A.E. Santoro, B.B. Oakley, Ubiquity and diversity of ammonia-oxidizing archaea in water columns and sediments of the ocean, Proc. Natl. Acad. Sci. USA. 102 (2005) 14683–14688. https://doi.org/10.1073/pnas.0506625102.

[5] J.H. Rotthauwe, K.P. Witzel, W. Liesack, The ammonia monooxygenase structural gene amoA as a functional marker: Molecular fine-scale analysis of natural ammonia-oxidizing populations, Appl. Environ. Microb. 63 (1997) 4704–4712. https://doi.org/10.1128/aem.63.12.4704-4712.1997.

[6] W. Walters, E.R. Hyde, D. Berg-Lyons, G. Ackermann, G. Humphrey, A. Parada, et al, Improved bacterial 16S rRNA gene (V4 and V4-5) and fungal internal transcribed spacer marker gene primers for microbial community surveys, mSystems 1 (2016) e00009–00015. https://doi.org/10.1128/mSystems.00009-15.
